# A Case of Highly Recurrent DFSP: Treatment Dilemmas and Considerations

**DOI:** 10.1155/crdm/6640596

**Published:** 2025-03-14

**Authors:** Ty Theriot, Christopher Haas

**Affiliations:** Department of Dermatology, Louisiana State University Health Sciences Center, New Orleans, Louisiana, USA

**Keywords:** CD34, dermatofibrosarcoma protuberans, soft tissue tumor

## Abstract

Dermatofibrosarcoma protuberans (DFSP) is a rare, slow-growing soft tissue tumor affecting the dermis and subcutaneous tissues, with potential involvement of muscle and fascia. This case report describes a 68-year-old Caucasian male with a history of recurrent DFSP on the left cheek, initially excised 36 years ago, with multiple recurrences despite wide local excisions (WLEs), eventually requiring left orbital enucleation, presenting to the clinic with a 10-year history of a slow-growing lesion on the left temporal scalp. Examination revealed a 2 cm flesh-colored, firm nodule, which biopsy confirmed as DFSP. Despite two subsequent WLEs, positive margins persisted. The patient refused further surgical intervention and was referred for imatinib and radiation therapy, which he also declined. MRI revealed additional nodules near the left zygomatic arch and sternocleidomastoid. DFSP is diagnosed via biopsy, often confirmed with CD34 immunohistochemistry. Optimal treatment is Mohs micrographic surgery (MMS), but WLE is also used. The recurrence rate is high, especially in head and neck locations. This case underscores the necessity for multidisciplinary management and highlights the critical role of thorough physical and histopathologic examinations. Close clinical follow-up is essential due to the high recurrence risk within the first three years post-treatment. This report emphasizes the importance of early detection and comprehensive care strategies to manage DFSP effectively.

## 1. Introduction

Dermatofibrosarcoma protuberans (DFSP) is a rare soft tissue tumor involving the dermis, subcutaneous fat, and sometimes muscle and fascia. DFSP usually presents as a slow-growing, firm plaque located over the trunk (50%), extremities (35%), or head and neck (15%); albeit rarely, the neoplasm may arise over the vulva or scrotum [1]. Cause remains poorly understood; however, studies have implicated a potential related chromosomal translocation resulting in the fusion protein COL1A1-PDGFB leading to overproduction of platelet-derived growth factor (PDGF). Diagnosis is made via skin biopsy [2]. Optimal treatment involves Mohs micrographic surgery (MMS), although frequently it is treated with wide local excision (WLE). Imatinib is a current FDA-approved treatment for adults with unresectable, recurrent, or metastatic DFSP. The chance of metastasis is low; however, rate of recurrence is high. Age older than 50 is a risk factor of recurrence. Recurrence rate when treated with WLE is 7.3% compared to 1% with MMS. With local recurrence being most common in the first three years post-excision, close clinical follow-up is recommended every three to six months during this time and annually afterward [3]. In this case, we present a patient with highly recurrent, unusual DFSP of the head and neck with local destruction despite multiple WLEs.

## 2. Case Presentation

**Figure 1:**
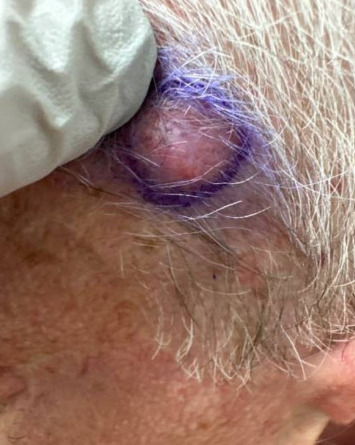
Clinical image displaying a 2 cm flesh-colored, firm tumor to the left fronto-temporal scalp with polymorphous vessels.

**Figure 2:**
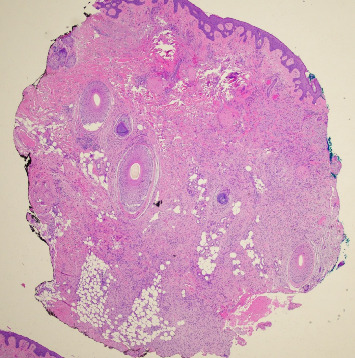
Hematoxylin and eosin stained specimen displaying spindle-shaped tumor cells arranged in a storiform or woven pattern, parallel to the epidermal surface, with little pleomorphism and scant cytoplasm.

**Figure 3:**
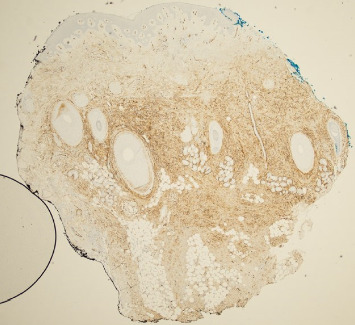
CD34 positive staining visualized in over 80% of the lesion.

A 68-year-old male with Fitzpatrick type II skin and a history of DFSP of the left cheek presented to our academic dermatology clinic reporting a ten-year history of a slow-growing lesion to the left temporal scalp. His initial excision was 36 years ago with failed pectoralis flap requiring left orbital enucleation, experiencing recurrences to the area every three to four years thereafter and not having consistent follow-up since his last recurrence. Physical exam at presentation revealed a 2 cm flesh-colored, firm nodule to the left scalp with polymorphous vessels (Figure 1). A shave biopsy was performed, and pathology showed spindle-shaped tumor cells arranged in a storiform or woven pattern, parallel to the epidermal surface, with little pleomorphism and scant cytoplasm consistent with DFSP (Figures 2 and 3). Patient was referred to otolaryngology, evaluated, and underwent two subsequent WLEs (1 cm margin at first excision followed by 4 mm margins from previous wound at second excision), still with positive margins. Patient refused further excisions and was referred to hematology/oncology and radiation oncology who recommended imatinib and radiation therapy, which patient refused. Magnetic resonance imaging of the face with and without contrast post-excision showed a 0.5 cm ovoid enhancing nodule in the superficial soft issues immediately caudal to the left zygomatic arch in addition to a 1.5 cm enhancing nodule overlying the left sternocleidomastoid.

## 3. Discussion

DFSP is a rare soft tissue tumor involving the dermis, subcutaneous layer, and sometimes muscle and fascia. Presentation typically involves a slow-growing, firm plaque with distribution to either the trunk (50%), extremities (35%), or head and neck (15%). This patient's presentation is noteworthy due to its atypical appearance on physical exam, origin in one of the less common anatomic regions of the body for DFSP, as well as its unusually high rate of recurrence and locally aggressive behavior. Diagnosis can often be made with hematoxylin and eosin staining. Confirmatory immunohistochemistry testing should take place in all patients, as 80%–100% of cases stain positive for CD34. Optimal treatment involves MMS, although sometimes it can be treated with WLE [1–3]. Recent studies have demonstrated that MMS has superior disease-specific survival when compared to WLE [4]. Imatinib is a current FDA-approved treatment for adults with unresectable, recurrent, or metastatic DFSP [5]. The chance of metastasis is low; however, the rate of recurrence is high. With local recurrence being most common in the first three years after treatment, close clinical follow-up is recommended every three to six months during this time and annually afterward [3].

While DFSP of the head and neck accounts for a small percentage of all cases, they have a high chance of local recurrence (≤ 56%). Depending on location, management should include a multidisciplinary team with head and neck surgeons, dermatologic surgeons, surgical oncologists, plastic surgeons, neurosurgeons, radiation oncologists, and medical oncologists. Unfortunately, no significant risk factors have been identified [1–3]. Prior studies have shown that tumor depth can predict recurrence in primary DFSP, whereas margin status can predict recurrence in locally recurrent DFSP [6]. Treatment for locally-recurrent DFSP includes MMS as well as margin control with frozen sectioning if performed by otolaryngology under general anesthesia. Surgical excision is the standard of care for this tumor, although multiple recurrences are usually discussed in tumor board by multiple specialists. Positive margins occur commonly, especially if surgical margins are conservative, even with WLE. MMS has been shown to provide superior outcomes for these recurrences as better margin control is attained.

The differential diagnosis initially considered other conditions with similar presentations, such as epithelioid sarcoma, atypical fibroxanthoma, and basal cell carcinoma (BCC). Each of these conditions has distinct clinical and histopathologic features, but they were ruled out based on the patient's presentation and diagnostic workup.

Epithelioid sarcomas—rare soft issue sarcomas that mimic granulomatous disease, carcinoma, and synovial sarcoma—were considered to be one possibility. They are more frequently found in males, and specifically in younger males with presentation on the distal upper extremity. Patients usually present with a mass or swelling in the absence of other signs or symptoms. Definitive diagnosis involves nodular/lobular architecture with central areas of necrosis surrounded by eosinophilic cytoplasm, with appearance mimicking granulomatous diseases. Treatment involves surgical excision, radiation, and palliative measures for metastatic disease [6]. Despite similarities on physical exam, this patient's age and location of lesion make this diagnosis less likely.

Another consideration was atypical fibroxanthoma which refers to a rare, low-grade superficial sarcoma that frequently presents as a red nodule or plaque. Ultraviolet (UV) light appears to play a role in development, as it often appears in sun-exposed areas of elderly, Caucasian patients. Physical examination often displays a well-circumscribed red or pink nodule or plaque that can ulcerate, crust, or scale. Definitive diagnosis involves biopsy revealing a dermal tumor with pleomorphism, atypical mitotic figures, and a spindly architecture on histopathology. With low risk of metastasis, surgical excision is often the treatment of choice [7]. Location and presentation of this patient's lesion are consistent with the pattern of atypical fibroxanthoma; however, the biopsy specimen showed no pathologic signs of this diagnosis.

As the most common cutaneous malignancy, BCC was part of the differential diagnosis. Presentation usually arises on sun-damaged skin with flesh- or pink-colored, pearly papules with overlying ulceration or telangiectatic vessels. Etiology typically derives from exposure to UV light, specifically the UVB wavelengths. Diagnosis is made via histopathology and reveals islands/nests of basaloid cells—each composed of basophilic nuclei—palisading at the periphery in a haphazard arrangement in the center of islands. Treatment of BCC is usually surgical, but some forms of BCC are suited for medical/radiation treatment [8]. Examination of this patient's lesion was very similar to BCC; however, histopathology ruled out this diagnosis.

In conclusion, this case highlights the importance of scheduled follow-up and multidisciplinary care in the management of recurrent DFSP, as these have been shown to have propensity for both further recurrence as well as deeper invasion into structures such as fascia, especially if not excised with wide margins. This has been demonstrated even on the head and neck regions with surgical margins ranging from 2 cm to 4 cm. DFSP patients are at increased risk of different visceral/hematologic malignancies which further emphasizes the importance of thorough physical and histopathologic examination in caring for patients with highly recurrent diseases [9]. Treatment begins with either margin control with MMS or WLE with margins recommended from 3 to 5 cm as well as MMS. MMS, “slow Mohs” variation of MMS, or intraoperative frozen margin evaluation likely offers lesser chance of recurrence of these tumors, especially on the head and neck region.

## Data Availability

The data that support the findings of this study are openly available in public repositories with DOIs as listed in the references section.
